# *Enterococcus faecalis* Encodes an Atypical Auxiliary Acyl Carrier Protein Required for Efficient Regulation of Fatty Acid Synthesis by Exogenous Fatty Acids

**DOI:** 10.1128/mBio.00577-19

**Published:** 2019-05-07

**Authors:** Lei Zhu, Qi Zou, Xinyun Cao, John E. Cronan

**Affiliations:** a College of Life Sciences, Shandong Agricultural University, Taian, Shandong, China; b Department of Microbiology, University of Illinois at Urbana-Champaign, Urbana, Illinois, USA; c Department of Biochemistry, University of Illinois at Urbana-Champaign, Urbana, Illinois, USA; University of Georgia; St. Jude Children's Research Hospital; University of California, San Diego

**Keywords:** FabT, phospholipids, acyl carrier protein, fatty acid synthesis, transcriptional regulation

## Abstract

Acyl carrier proteins (ACPs) play essential roles in the synthesis of fatty acids and transfer of long fatty acyl chains into complex lipids. The Enterococcus faecalis genome contains two annotated *acp* genes, called *acpA* and *acpB*. AcpA is encoded within the fatty acid synthesis (fab) operon and appears essential. In contrast, AcpB is an atypical ACP, having only 30% residue identity with AcpA, and is not essential. Deletion of *acpB* has no effect on E. faecalis growth or *de novo* fatty acid synthesis in media lacking fatty acids. However, unlike the wild-type strain, where growth with oleic acid resulted in almost complete blockage of *de novo* fatty acid synthesis, the *ΔacpB* strain largely continued *de novo* fatty acid synthesis under these conditions. Blockage in the wild-type strain is due to repression of *fab* operon transcription, leading to levels of fatty acid synthetic proteins (including AcpA) that are insufficient to support *de novo* synthesis. Transcription of the *fab* operon is regulated by FabT, a repressor protein that binds DNA only when it is bound to an acyl-ACP ligand. Since AcpA is encoded in the *fab* operon, its synthesis is blocked when the operon is repressed and *acpA* thus cannot provide a stable supply of ACP for synthesis of the acyl-ACP ligand required for DNA binding by FabT. In contrast to AcpA, *acpB* transcription is unaffected by growth with exogenous fatty acids and thus provides a stable supply of ACP for conversion to the acyl-ACP ligand required for repression by FabT. Indeed, *ΔacpB* and *ΔfabT* strains have essentially the same *de novo* fatty acid synthesis phenotype in oleic acid-grown cultures, which argues that neither strain can form the FabT-acyl-ACP repression complex. Finally, acylated derivatives of both AcpB and AcpA were substrates for the E. faecalis enoyl-ACP reductases and for E. faecalis PlsX (acyl-ACP; phosphate acyltransferase).

## Abstract (precis)

AcpB homologs are encoded by many, but not all, lactic acid bacteria (*Lactobacillales*), including many members of the human microbiome. The mechanisms regulating fatty acid synthesis by exogenous fatty acids play a key role in resistance of these bacteria to those antimicrobials targeted at fatty acid synthesis enzymes. Defective regulation can increase resistance to such inhibitors and also reduce pathogenesis.

## INTRODUCTION

Fatty acid synthesis (FAS) is a key metabolic pathway that provides precursors for the formation of cellular membranes in mammals, plants, fungi, and bacteria (1). Moreover, the fatty acid synthesis pathway allows diversion of intermediates to other functional molecules such as the vitamins biotin and lipoic acid (2) plus various bacterial signaling molecules (3). Fatty acid synthesis in bacteria, mitochondria, and plant plastids is catalyzed by a set of discrete enzymes that are collectively known as the type II (FAS II) system (1). Acyl carrier proteins (ACPs) play central roles in the synthesis of fatty acids biosynthesis and their transfer into membrane lipids (1, 4). In *Firmicutes* bacteria, ACPs also play important roles in the uptake and utilization of exogenous fatty acids by the FakA/FakB pathway (5).

**FIG 1:**
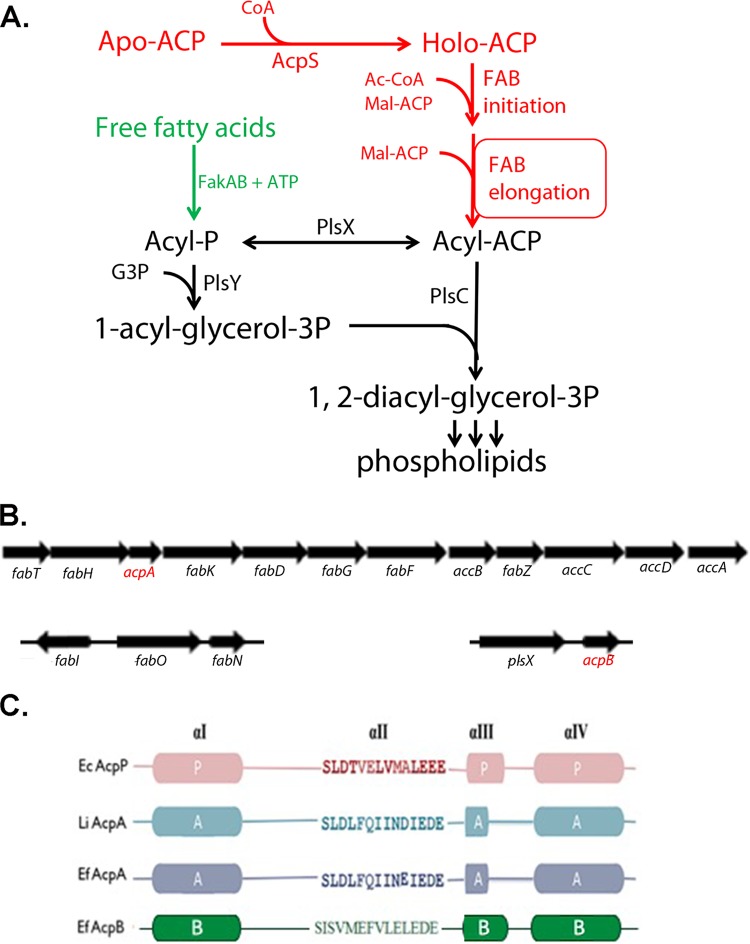
Enterococcus faecalis phospholipid synthesis. (A) Functions of ACP in phospholipid synthesis; (B) the genomic locations of the *acpA* and *acpB* genes in E. faecalis. The *fab* genes and *plsX* are also shown. (C) Sequence alignments of ACPs, showing the differences in helix II residues. The serine residue at the beginning of the helix carries the prosthetic group (the other three helices are given in cartoon form): EcAcpP, E. coli ACP; LiAcpA, L. lactis ACP; EfAcpA, E. faecalis AcpA; EfAcpB, E. faecalis AcpB. The *acpA* and *acpB* genes are present in all currently available E. faecalis genomes. The structure of AcpB has been determined by nuclear magnetic resonance approaches and was reported previously to have the conserved ACP four-helix configuration and to be unusually stable to high temperatures (32). Abbreviations are as follows: Ac-CoA, acetyl-CoA; Mal-ACP, malonyl-ACP.

In Staphylococcus aureus, exogenous fatty acids are phosphorylated by a complex of a fatty acid kinase (FakA) and a fatty acid binding protein (FakB) (5). The acyl-phosphates formed are either used by the PlsY glycerol-3-phosphate acyltransferase in the first acylation step of phospholipid synthesis or converted to acyl-ACPs by the PlsX acyl-ACP:phosphate acyltransferase (5, 6). The acyl-ACPs so generated may be either elongated by FAS II or used to complete the synthesis of phosphatidic acid by transfer of the acyl group to position 2 of 1-acyl-*sn*-glycerol-3-phosphate catalyzed by the PlsC 1-acyl-*sn*-glycerol-3-phosphate acyltransferase (Fig. 1A) (5, 6).

Escherichia coli ACP (called AcpP), the most thoroughly studied member of the ACP family, is an abundant, small, and negatively charged protein that is essential for growth (7–9). Prior work showed that expression of the ACPs from a diverse set of bacteria could replace the function of E. coli ACP in lipid biosynthesis (8). Of the bacteria tested, only the Enterococcus faecalis and Lactococcus lactis AcpAs failed to support growth of an E. coli *acpP* mutant strain (8). Construction of chimeric proteins containing the E. coli AcpP and L. lactis AcpA sequences showed that specific protein sequences located largely in helix II were incompatible with an E. coli lipid synthesis enzyme(s) (9).

The AcpA of L. lactis rather than that of E. faecalis was used for the prior ACP chimera studies (9) because the genome of the latter bacterium contained a gene encoding a putative second ACP of unknown function. This E. faecalis gene (locus tag EF3111), called *acpB* in this report, appears to be cotranscribed with the *plsX* gene of phospholipid synthesis, and its location implies a role in fatty acid metabolism rather than a related pathway (e.g., polyketide synthesis). However, *acpB* encodes an amino acid chain that is only 30% identical to that of AcpA and, surprisingly, shows higher identities to the AcpPs of E. coli (47%) and Bacillus subtilis (54%) than to AcpA (Fig. 1B and C). Most strikingly, AcpB has helix II residues that differ markedly from those of the E. faecalis and L. lactis AcpAs, including the eight residues downstream of the putative site of 4′-phosphopantetheinyl moiety attachment (Fig. 1B). Since many of these residues play important roles in ACP function in E. coli (8–11), this raised the issues of whether or not AcpB becomes modified with 4‘-phosphopantetheine and the physiological role of the protein.

E. faecalis incorporates exogenous fatty acids as well as *de novo* synthesized fatty acids for assembly of its membrane phospholipids (12, 13). Our goal was to determine the functions of the two ACPs in these pathways. It seemed clear that *acpA* encoded the canonical ACP of fatty acid synthesis because *acpA* is encoded in the *fab* operon and is cotranscribed with the upstream and downstream genes (12). Moreover, E. faecalis AcpA is 64% identical to the sole L. lactis ACP. Hence, it seemed clear that AcpA performed the “heavy lifting” in *de novo* fatty acid biosynthesis whereas AcpB seemed likely to have a function other than fatty acid synthesis given its divergent sequence. As noted above, many but not all *Lactobacillales* spp. have an *acpB* homolog encoded immediately downstream of PlsX. A notable exception is L. lactis.

## RESULTS

### Analysis of E. faecalis *acpA* and *acpB* genes *in vivo*.

**FIG 2:**
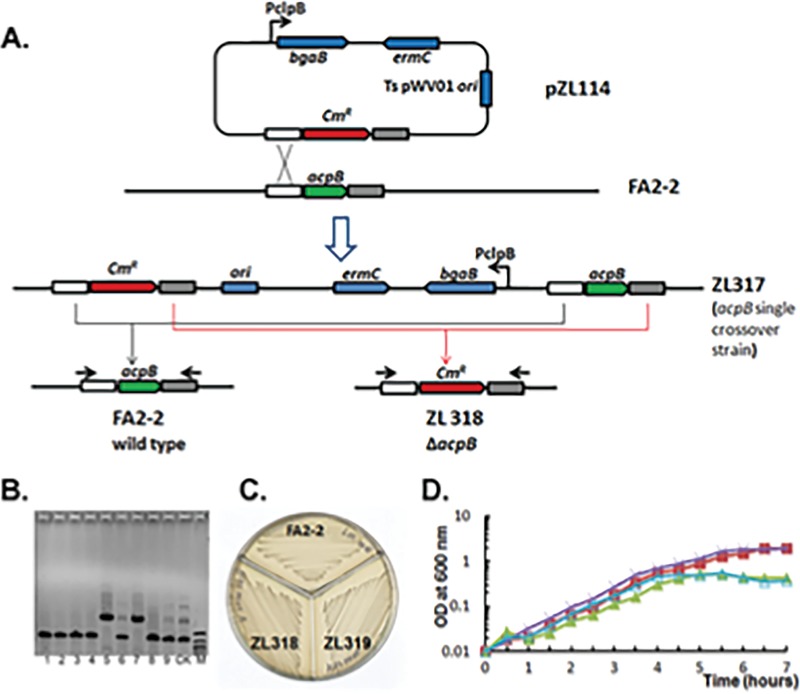
Construction and characterization of E. faecalis *acpB* gene (*ΔacpB*) deletion strains. (A) The strategy for construction of the E. faecalis *ΔacpB* strain paralleled that reported previously (13) except that the entire *acpB* coding sequence was replaced with a chloramphenicol resistance (Cm^r^) “stuffer” fragment which facilitated PCR analysis of recombinant candidates. (B) Characterization of the E. faecalis *ΔacpB* strains by PCR. Lanes 1 to 9 represent the PCR products amplified using candidate genomic DNAs as the template. The strains analyzed in lanes 5 and 7 were named ZL318 and ZL319, respectively; the lane marked “CK” contained the PCR product obtained using strain E. faecalis FA2-2 genomic DNA as the template. M, DNA ladder. DNA sequencing of the strain ZL318 PCR product confirmed that the construction had proceeded as planned. (C) Growth phenotype of E. faecalis *ΔacpB* strains on M17 medium plates. (D) Growth curves of E. faecalis FA2-2 and ZL318 strains in M17 medium with or without oleate. Symbols: ×, ZL318 without oleate; ▪, FA2-2 without oleate; ▵, FA2-2 with oleate; ▲, ZL318 with oleate. The growth curves of the two strains grown with or without of oleate supplementation were superimposable. Note that a sequence of >1,500 bp separates the *acpB* gene from the next annotated gene located downstream, which is the first gene of a peptide transport operon.

We first asked if both *acpA* and *acpB* were essential genes. As described above, it seemed that *acpA* might be essential. However, in prior work we constructed a strain lacking both enoyl reductases (FabI and FabK) that grew well in the presence of oleate (12), implying that an *acpA* deletion strain might have the same phenotype. However, despite numerous attempts, we were unable to construct an *acpA* deletion strain. In contrast, using the same protocols, we readily obtained *acpB* (Δ*acpB*) deletion strains that grew well without exogenous oleic acid (Fig. 2). This raised the issue of whether or not *acpB* was a pseudogene that was not expressed or that expressed a protein that could not be modified with 4′-phosphopantetheine, which represents the litmus for identification of ACPs (14).

**FIG 3:**
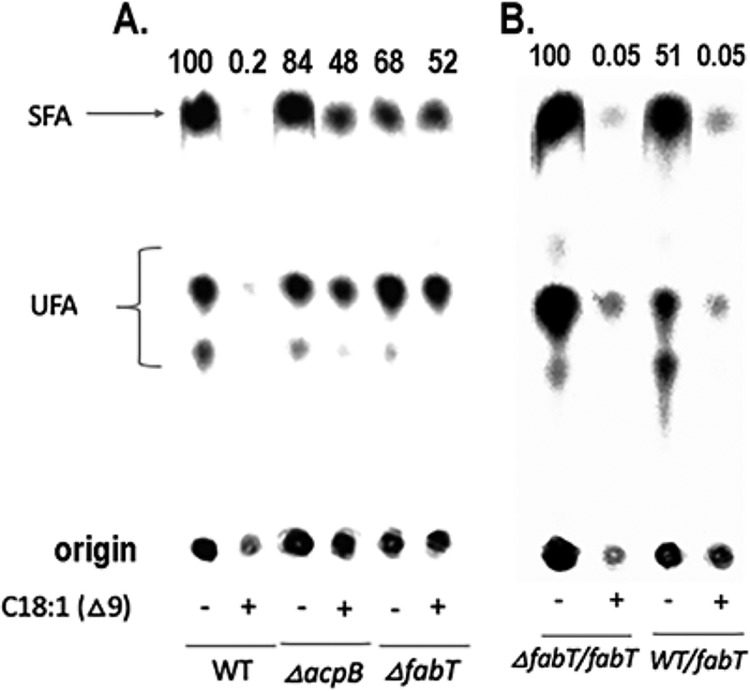
*De n*ovo fatty acid synthesis in E. faecalis strains grown in the absence or presence of oleate. (A) The phospholipid fatty acids of the wild-type, *ΔacpB*, and *ΔfabT* strains were labeled with [1-^14^C]acetate. The lanes are designated below the autoradiograms, and the numbers above the lanes give the relative incorporation values obtained with the wild-type strain grown without oleate, which was given a value of 100. SFA, saturated fatty acids; UFA, unsaturated fatty acids. (B) Strains carrying a plasmid encoding FabT were labeled as described above. The plasmid (designated *fabT*) restored regulation to the *ΔfabT* strain (lanes marked *ΔfabT*/*fabT*) but did not increase repression in the wild-type strain (lanes marked WT/*fabT*), which was given a value of 100. The lanes are designated below the autoradiograms, and the numbers above the lanes represent the incorporation values obtained. Note that oleate was used rather than palmitate or myristate because the saturated fatty acids inhibited growth of E. faecalis, presumably because they blocked synthesis of essential unsaturated fatty acids. Relative to cultures grown without oleate in five biological replicates of each experiment, growth with oleate decreased [1-^14^C]acetate incorporation into the phospholipids of the wild-type strain by between 20-fold and 70-fold whereas, in parallel experiments performed with the *ΔacpB* strain, oleate growth decreased [1-^14^C]acetate incorporation by 1.8-fold to 2.3-fold. In the *ΔfabT* strain, oleate growth decreased [1-^14^C]acetate incorporation by 1.9-fold to 2.4-fold or (in one experiment) increased incorporation by 0.2-fold to 2-fold.

In the hope of uncovering a phenotype for the Δ*acpB* mutation, we tested the effects of growth in the presence of oleic acid on *de novo* fatty acid synthesis because several other members of the *Lactobacillales* group of bacteria are known to shut down *de novo* fatty acid synthesis when grown with exogenous unsaturated fatty acids (5, 14, 15). Indeed, oleic acid supplementation of the medium of the E. faecalis wild-type (WT) strain almost completely blocked *de novo* synthesis from [1-^14^C]acetate (a ∼50-fold decrease) whereas growth with oleate had only a small (∼2-fold) effect in the *ΔacpB* strain (Fig. 3A). Although this phenotype indicated that *acpB* was a functional gene, the more important finding was that these results strongly resembled the phenotype reported for *ΔfabT* strains of the related bacterium Streptococcus pneumoniae (14) in which growth of the wild-type strain with oleic acid resulted in essentially complete blockage of fatty acid synthesis (15). This blockage is mediated by a repressor called FabT that binds the operator sites of the fatty acid biosynthesis operon and thereby represses transcription of the *fab* genes (14). However, FabT binds DNA only when complexed with acyl-ACP species that have long-chain acyl moieties (16). E. faecalis encodes a putative FabT that is 51% identical to that of S. pneumoniae. The putative *fabT* gene is encoded in a fatty acid synthesis operon (Fig. 1) that is very similar to the S. pneumoniae operon and is cotranscribed with the downstream *fabH* and *acpA* genes (16). To compare the activity of the putative E. faecalis FabT with that of S. pneumoniae, we deleted *fabT* from the genome and tested the response of the *ΔfabT* strain to oleate supplementation. We found that oleate supplementation essentially abolished *de novo* fatty acid synthesis in the wild-type strain (a 20-fold decrease) whereas the *ΔfabT* strain showed only a modest (2-fold) decrease such as was seen in the *ΔacpB* strain (Fig. 3B). Introduction of a *fabT*-harboring plasmid into the *ΔfabT* strain restored repression by oleate supplementation, whereas introduction of the *fabT* plasmid into a wild-type strain gave a modest (perhaps 2-fold) increase in repression (Fig. 3B). Therefore, E. faecalis FabT functions in a manner similar to that seen with S. pneumoniae FabT. Moreover, the effects of exogenous oleate on *de novo* fatty acid synthesis were essentially identical in the *ΔfabT* and Δ*acpB* strains. That is, loss of AcpB mimicked loss of FabT.

**FIG 4:**
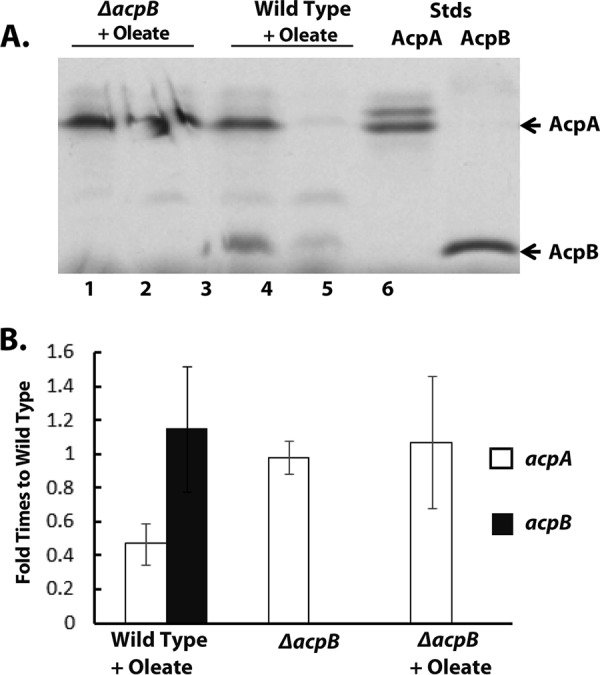
Expression of AcpA and AcpB and *acp* gene transcription in E. faecalis. (A) E. faecalis strains were grown in AC medium with or without 100 μM sodium oleate. The cells were lysed, and the supernatants were loaded onto a 5-ml Hitrap DEAE column. The bound proteins were eluted with 25 mM K-MES (pH 6.1) containing 2 M KCl solution. These samples were heated at 75°C, deacylated by treatment with DTT at pH 10.0 to cleave the thioester bonds, and then dialyzed. Protein from each extract was then incubated with E. faecalis *holo*-ACP synthase (AcpS) to convert any *apo* protein to the *holo* form followed by V. harveyi acyl-ACP synthetase-catalyzed conversion to [1-^14^C]octanoyl-ACPs. The products were then analyzed by 2 M urea–18% PAGE followed by autoradiography. The details of the treatments and enzymatic conversions are given in Materials and Methods. The relative intensities of the bands are given below the gel. Although the *ΔacpB* plus oleate lane suffered cracking during drying of the gel, this did not interfere with quantitation because no gel was lost. The AcpA and AcpB standards (Stds) were obtained by expression in E. coli. (B) RT-qPCR analysis of the relative transcription levels of E. faecalis *acpA* and *acpB* in wild-type and *ΔacpB* strains grown with or without oleate. The levels of *acpA* and *acpB* transcripts are given relative to the expression level of the wild-type strain grown in the absence of oleate. The data are from three biological repeats of the experiments. Open columns, *acpA* transcription levels; solid columns, *acpB* transcription levels. No *acpB* transcripts were detected in the *ΔacpB* strain.

The phenotype of the Δ*acpB* strain demonstrated that *acpB* was expressed. However, it remained unclear if AcpB could be modified with 4’-phosphopantetheine; the criterion for a *bona fide* ACP (17). Assessing modification of AcpB and AcpA was problematical since both proteins are expressed at much lower levels than the AcpPs of E. coli and B. subtilis studied previously. The direct assays used in those bacteria lacked the sensitivity needed for E. faecalis analysis; thus, we resorted to a protocol in which AcpA and AcpB were enriched from E. faecalis cell extracts by ion-exchange chromatography and assayed by acylation with [1-^14^C]octanoate. The efficiency of the ion exchange enrichment protocol was demonstrated by the efficient purification of AcpA and AcpB expressed in E. coli (see below). The low E. faecalis ACP levels and the presence of contaminating proteins precluded standard PAGE gel analyses; thus, we turned to a specific radiochemical assay. The ion exchange fractions were first treated with a strong reducing agent at a high denaturing pH to cleave the thioester bonds of any acyl-ACPs present. After dialysis, the deacylated protein fractions were treated with E. faecalis AcpS and coenzyme A (CoA) (to convert any *apo* ACPs to the *holo* form) and then modified with Vibrio harveyi AasS (acyl ACP synthetase) (18), ATP, and [1-^14^C]octanoate to give [1-^14^C]octanoyl-ACPs, which, following electrophoresis, were quantitated by radioactive counting. AasS activity on both AcpA and AcpB had previously been validated using AcpA and AcpB expressed in E. coli after conversion to their *holo* forms by use of the E. faecalis phosphopantetheinyl transferase (EF0848; see below). These experiments showed that E. faecalis AcpB, like AcpA, was modified with phosphopantetheine and could be acylated. The identity of the AcpB band was confirmed by its absence in the *ΔacpB* strain (Fig. 4A). Moreover, deletion of *acpB* in strain ZL318 blocked the oleate-engendered repression of *acpA* expression at the protein level (Fig. 4A) as expected from the *de novo* fatty acid synthesis labeling experiments (Fig. 3). In two biological repeats of this experiment, the levels of AcpB relative to AcpA ranged from 27% to 47% in cultures of the wild-type strain grown without oleate. We attribute this variation to the large number of manipulations involved in the analysis. In both experiments, however, growth with oleate severely decreased the levels of AcpA but had no effect on AcpB levels. Given the indirect and multistep nature of this experimental approach, we turned to transcriptional analyses. Expression of the E. faecalis *acpA* and *acpB* genes was measured at the transcriptional level by real-time reverse transcription-quantitative PCR (RT-qPCR). RNA was extracted and reverse transcribed, and the cDNA concentrations were normalized using the 16S RNA gene as the internal reference. The E. faecalis *acpA* and *acpB* gene expression levels in the wild-type strain (FA2-2) grown in M17 medium were defined as expression levels of 1.0. In agreement with the protein data obtained in three biological repeats of the experiments, the levels of *acpA* transcripts were markedly decreased in oleate-grown cultures whereas those of *acpB* transcripts were unaffected by growth with oleate (Fig. 4B).

### E. faecalis *acpA* and *acpB* cannot replace the functions of E. coli *acpP in vivo*.

**FIG 5:**
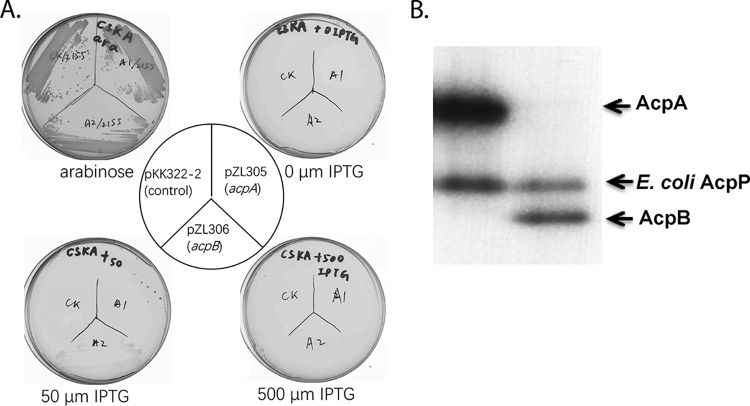
Expression of E. faecalis *acpA* and *acpB* fails to permit growth of E. coli strain CY2155 with conditional *acpP* expression. (A) Growth of derivatives of E. coli *sfp*-expressing *acpP* mutant strain CY2155 expressing either E. faecalis AcpA or E. faecalis AcpB. The strains carried plasmids harboring E. faecalis *acpA* or E. faecalis *acpB* (pZL305 or pZL306, respectively) or the vector pKK233-2. The CY2155 transformants were incubated on LB medium without or with 0.02% arabinose (the presence of arabinose is permissive for conditional *acpP* expression [9]), 50 μM IPTG, or 500 μM IPTG. (B) Expression of E. faecalis AcpA or AcpB in E. coli CY2211, a *ΔpanD* strain expressing *sfp*. The CY2211 transformants were grown with β-[2,3-^3^H]alanine in M9 glycerol medium with arabinose. The strains carried plasmids harboring E. faecalis *acpA* or E. faecalis *acpB* (pZL305 or pZL306, respectively). Lane 1, expression of ACPs in CY2211 transformed with pZL305; lane 2, expression of ACPs in CY2211 transformed with pZL306.

Previous work from this laboratory demonstrated that neither E. faecalis *acpA* nor L. lactis *acpA* could functionally replace E. coli *acpP* even when the promiscuous B. subtilis Sfp 4-phosphopantetheinyl transferase was provided to overcome the inability of E. coli AcpS to modify these *apo* ACPs (8, 9). However, since the AcpB sequence was more similar to those of the ACPs of E. coli and B. subtilis, we tested the ability of AcpB expression to replace E. coli AcpP. This was tested in the presence of Sfp because expression of AcpB in E. coli gave only the *apo* form, indicating that the E. coli AcpS 4′-phosphopantetheinyl transferase was inactive on this ACP. As expected, AcpA expression completely failed to restore growth (Fig. 5A). However, AcpB expression gave a faint haze of growth that was dependent on the presence of the IPTG (isopropyl-β-d-thiogalactopyranoside) inducer but only at the lower inducer concentration of 50 μM (Fig. 5A). A higher IPTG concentration inhibited growth, suggesting that high-level AcpB expression is toxic to E. coli. A straightforward explanation for the weak growth given by AcpB expression would be poor expression and/or 4-phosphopantetheine modification of the protein. To test this possibility, we labeled expression strains with β-[2,3-3H]alanine in the presence of IPTG. The “ACP-testing” CY2211 strain carries a *panD* deletion and thus requires the CoA precursor β-alanine (or panthothenate) for growth (19). Since CoA is the source of ACP 4-phosphopantetheine moieties, radioactive β-alanine labels only 4-phosphopantetheine-modified proteins (19). This labeling showed that both E. faecalis AcpA and AcpB were modified and expressed at levels higher than the host AcpP and were hence expected to be sufficient to support growth of strain CY2155 (Fig. 5B).

### Expression and purification of E. faecalis AcpA and AcpB proteins and their activity with two fatty acid synthetic enzymes.

**FIG 6:**
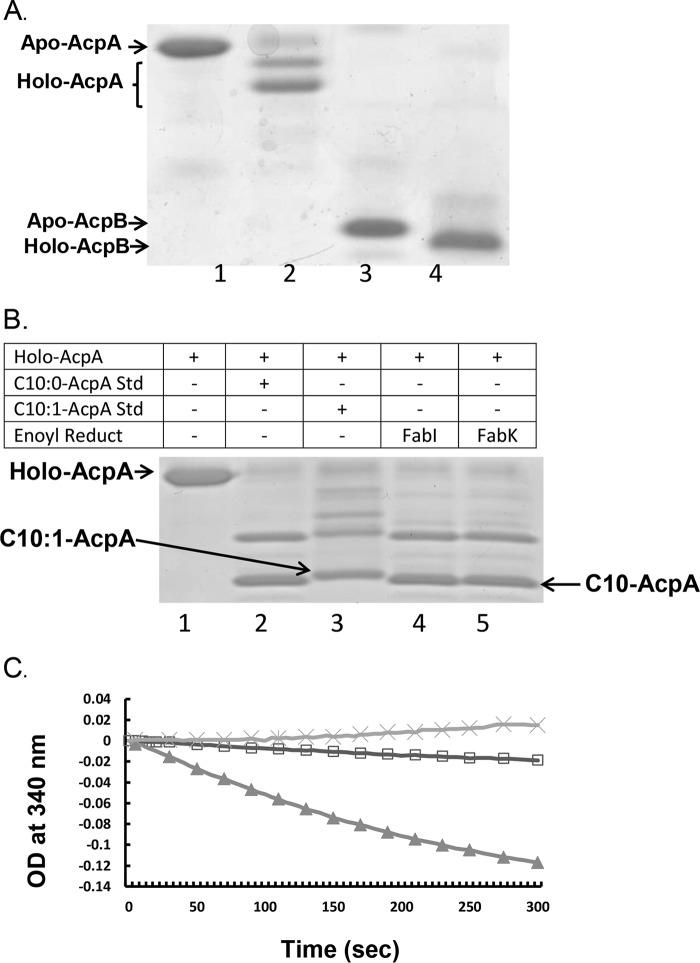
Properties of the E. faecalis ACPs. (A) E. faecalis AcpS catalyzed conversion of E. coli-expressed *apo*-ACPs to *holo*-ACPs. Lane 1, *apo*-AcpA; lane 2, AcpS reaction with *apo*-AcpA to give *holo*-AcpA; lane 3, *apo*-AcpB; lane 4, AcpS reaction with *apo*-AcpB to give *holo*-AcpB. (B) E. faecalis enoyl-ACP reductase reactions of *trans*-2-decenoyl-AcpA. Lane 1, *holo*-AcpA standard; lane 2, AasS-synthesized decanoyl-AcpA standard; lane 3, AasS-synthesized *trans*-2-decenoyl-AcpA standard; lane 4, FabI reaction with *trans*-2-decenoyl-AcpA and NADH; lane 5, FabK reaction with *trans*-2-decenoyl-AcpA and NADH. (C) Reduction *of trans*-2-decenoyl-AcpB by the FabI or FabK E. faecalis enoyl-ACP reductase. The reactions were monitored by the decrease in NADH UV absorption at 340 nm. Symbols: ×, no-reductase control; ▵, FabK; □, FabI.

A remaining issue concerned the ability of AcpB to function in fatty acid synthesis. To approach this issue, the E. faecalis *acpA* and *acpB* genes were expressed in E. coli. As expected, the resulting small, acidic proteins were extremely soluble and readily purified by ion-exchange chromatography. In the case of AcpA, contamination with E. coli ACP was a problem; thus, AcpA was purified from a strain of E. coli in which the chromosomal *acpP* gene carries a C-terminal streptavidin (Strep) tag sequence (S. Srinivas and J. E. Cronan, unpublished data). When applied to a Strep-Tactin column, the Strep-tagged E. coli ACP was bound quantitatively whereas AcpA passed through the column. Contaminating proteins that coeluted with AcpA were removed by ammonium sulfate precipitation (where ACPs are soluble). To prepare enzyme substrates, we purified the N-terminal hexahistidine-tagged versions of E. faecalis AcpS (*holo*-ACP synthase), the E. faecalis FabI and FabK enoyl-ACP reductases, and V. harveyi AasS (acyl ACP synthetase) proteins by nickel-chelate chromatography. AcpS was used to convert the *apo* proteins to the *holo* proteins required for AasS-catalyzed acylation (Fig. 6A). Upon gel electrophoresis (Fig. 4), two forms of AcpA were observed (Fig. 4). Electrospray mass spectrometry (MS) showed the two forms to have intact masses of 8,479.27 and 8,610.32, representing a mass difference of 131.05, which is the mass of a methionyl residue. Therefore, the 8,610.32 species, which is the major form in mass spectra, had retained the initiator methionine residue whereas the E. coli methionine aminopeptidase had removed that residue from the 8,479.27 species. The observed partial cleavage is consistent with the AcpA sequence where the second residue is valine. Met-Val sequences result in variable cleavage by methionine aminopeptidase (20). Note that overproduction of E. coli ACP in E. coli also results in incomplete initiator methionine removal due to titration of the methionine aminopeptidase (21) and that titration may also be a factor in AcpA processing.

Incubation of the *holo* proteins with AasS, ATP, and *trans*-2-decenoic acid resulted in *trans*-2-decenoyl-AcpA and *trans*-2-decenoyl-AcpB, the substrates used to assay function with the E. faecalis enoyl-ACP reductases FabI and FabK. In the case of *trans*-2-decenoyl-AcpA, this substrate was incubated with either FabI or FabK plus NADH followed by analysis by conformationally sensitive urea-PAGE. Although partial methionine aminopeptidase cleavage resulted in two different forms of *trans*-2-decenoyl-AcpA, both were reduced to decanoyl-AcpA by FabI and FabK (Fig. 6B).

Further assays indicated that FabI was more efficient in reduction of *trans*-2-decenoyl-AcpA than FabK (data not shown). PAGE did not separate *trans*-2-decenoyl-AcpB from the decanoyl-AcpB product. Therefore, the reductase reaction was followed by spectroscopic assay of NADH oxidation (decreased absorbance at 340 nm). FabK was more active than FabI (Fig. 6C), although FabI is the primary E. faecalis enoyl-ACP reductase (8, 9).

### Function of E. faecalis AcpA and AcpB in acyl group transfer between ACP and phosphate.

In addition to fatty acid synthesis, ACPs play key roles in acylation of *sn*-glycerol-3-phosphate to produce the early intermediates of phospholipid synthesis. In the *Lactobacillales* bacteria, both acyl-ACP and acyl-phosphate are required for synthesis of phosphatidic acid, the first fully acylated intermediate in phospholipid synthesis (5, 6). As described in the introduction, PlsX catalyzes acyl transfer between ACP and phosphate (Fig. 1A). The acyl chains can be derived either by *de novo* synthesis or by uptake from the medium. The inability of the *ΔacpB* deletion strain to repress AcpA expression in the presence of oleate (Fig. 3A) argued that AcpA and AcpB might differ in their oleoyl transfer activities. One possibility was that oleoyl transfer from oleoyl-phosphate, the first intermediate in oleate incorporation into phospholipid, to AcpB might be more efficient than transfer to AcpA. To assay the acyl transfer reactions *in vitro*, we purified an N-terminal hexahistidine-tagged version of E. faecalis PlsX and chemically synthesized myristoyl-phosphate (C14:0-PO_4_), palmitoyl-phosphate (C16:0-PO_4_), stearoyl-phosphate (C18:0-PO_4_), and oleyl-phosphate (C18:1-PO_4_). The same acyl chains (except oleyl-ACP, an inactive AasS substrate) were converted to acyl-ACPs using V. harveyi AasS.

**FIG 7:**
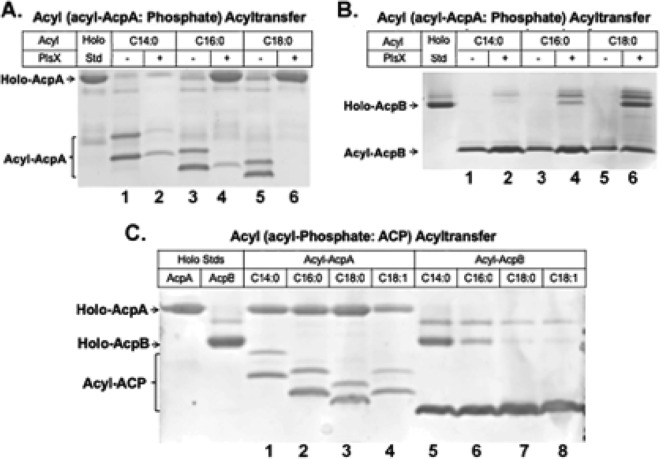
Function of AcpA and AcpB in generation of substrates for phospholipid acylation. (A) E. faecalis PlsX catalysis of transfer of acyl groups from acyl-AcpA to phosphate. For *holo*-AcpA, lanes 1 and 2, myristoyl-AcpA (C14:0-AcpA) minus or plus PlsX; lanes 3 and 4, palmitoyl-AcpA (C16:0-AcpA) minus or plus PlsX; lanes 5 and 6, stearoyl-AcpA (C18:0-AcpA) minus or plus PlsX. The *holo*-AcpA standard is given in the leftmost lane. (B) E. faecalis PlsX catalyzes transfer of acyl groups from acyl-AcpB to phosphate. Lanes 1 and 2, myristoyl-AcpB (C14:0-AcpB) minus or plus PlsX; lanes 3 and 4, palmitoyl-AcpB (C16:0-AcpB) minus or plus PlsX; lanes 5 and 6, stearoyl-AcpB (C18:0-AcpB) minus or plus PlsX. The *holo*-AcpB standard is given in the leftmost lane. (C) E. faecalis PlsX catalyzes transfer of acyl groups from acyl-phosphates to ACPs. Lanes 1 to 4, transfer of the stipulated acyl chains from AcpA to phosphate; lanes 5 to 8, transfer of the stipulated acyl chains from AcpB to phosphate. *holo*-ACP standards are given in the leftmost two lanes.

We first tested transfer of acyl groups from acyl-ACP to phosphate. Sequential addition of the purified components and incubation were followed by analysis by conformationally sensitive PAGE. In this assay, transfer to phosphate was followed by the decrease in acyl-ACP levels. Both AcpA and AcpB donated the acyl group to phosphate in the PlsX-catalyzed reaction (Fig. 7A and B). The acyl chain of stearoyl-AcpA was fully transferred to phosphate (Fig. 7A, lane 6), whereas myristoyl-ACP and palmitoyl-ACP showed less transfer (Fig. 7A, lanes 3 and 4). However, transfer of acyl group from AcpB to phosphate was much less efficient even when stearoyl-ACP was the substrate, indicating that the level of activity of AcpB in transfer in the ACP to phosphate by PlsX was considerably lower than that of AcpA (Fig. 7B).

In the presence of exogenous oleic acid, AcpB acts to potently repress transcription of the fatty acid synthesis operon (Fig. 3 and 4). Upon entering the E. faecalis cytosol, oleic acid would be converted to oleyl-phosphate, which would then be usable either for acylation of *sn*-glycerol-3-phosphate via the PlsY reaction or for conversion to acyl-ACP by PlsX (Fig. 1A) (5, 6). To test the activities of AcpA and AcpB as acyl acceptors from acyl-phosphate (the reverse of the reaction assayed as described above), we incubated PlsX with various acyl-phosphates and either AcpA or AcpB. Acyl-ACP formation was analyzed by PAGE. Acyl transfer to AcpA was markedly inefficient, whereas AcpB was fully converted into acyl-AcpB when stearoyl-phosphate (C18:0-PO4) or oleyl-phosphate (C18:1-PO4) was the acyl donor (Fig. 7C, lanes 7 and 8).

### Deletion of *acpB* renders E. faecalis deficient in fatty acid incorporation.

**FIG 8:**
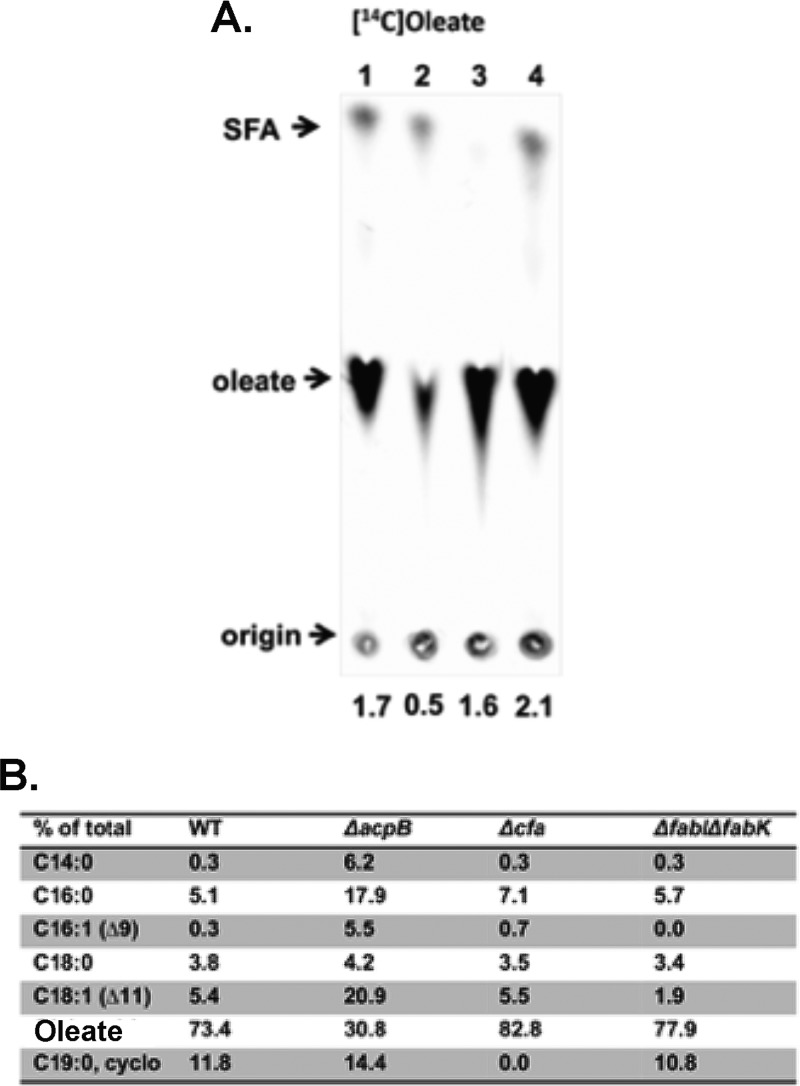
Analysis of phospholipid fatty acids in cells grown with exogenous oleate. (A) Incorporation of [1-^14^C]oleate into the phospholipids of the wild-type E. faecalis FA2-2 strain and derived mutant strains. Lane 1, [1-^14^C]oleate incorporation into the wild-type strain; lane 2, [1-^14^C]oleate incorporation into the *ΔacpB* strain; lane 3, [1-^14^C]oleate incorporation into the *Δcfa* strain; lane 4, [1-^14^C]oleate incorporation into the *ΔfabI ΔfabK* strain. The numbers below the origin give the incorporation values for each lane. In two biological replicates of this experiment, the level of [1-^14^C]oleate incorporation into the phospholipids of the *ΔacpB* strain was 2.7-fold to 3.4-fold lower than that incorporated by the wild-type strain. (B) GC-MS analysis of phospholipid fatty acid compositions of E. faecalis strains grown with oleate.

Incorporation of exogenous fatty acid into membrane phospholipids was directly tested by [1-^14^C]oleate labeling. These results showed that, relative to the levels seen with wild-type strains, oleate incorporation into the phospholipids of the Δ*acpB* strain was decreased by 3-fold to 4-fold (compare lane 1 of Fig. 8A to lane 2). In contrast, incorporation of [1-^14^C]oleate into phospholipids of the *ΔfabI ΔfabK* strain in which *de novo* fatty acid synthesis is totally blocked (12) was not significantly different from that seen with the wild-type strain (Fig. 8A lane 4). This was expected, since the wild-type strain synthesized almost no fatty acid when oleate was present in medium (Fig. 3A, lane 2). The accumulation of some labeled saturated fatty acid methyl ester in the phospholipids represented a puzzle. Deletion of the *cfa* gene showed this to be the cyclopropane derivative of oleate (*cis*-9,10-methylene octanoic acid). Cyclopropane fatty acyl phospholipid synthase (CFA) converts the double bonds of the unsaturated moieties of membrane phospholipids to saturated cyclopropane acids (22). The fatty acid components of the phospholipids of these strains grown with oleate were analyzed by gas chromatography-mass spectrometry (GC-MS) and showed that the level of oleate in the phospholipids of the Δ*acpB* strain was strongly decreased relative to those seen with the other three strains (Fig. 8B). (Note that oleate is not a natural E. faecalis fatty acid.)

## DISCUSSION

**FIG 9:**
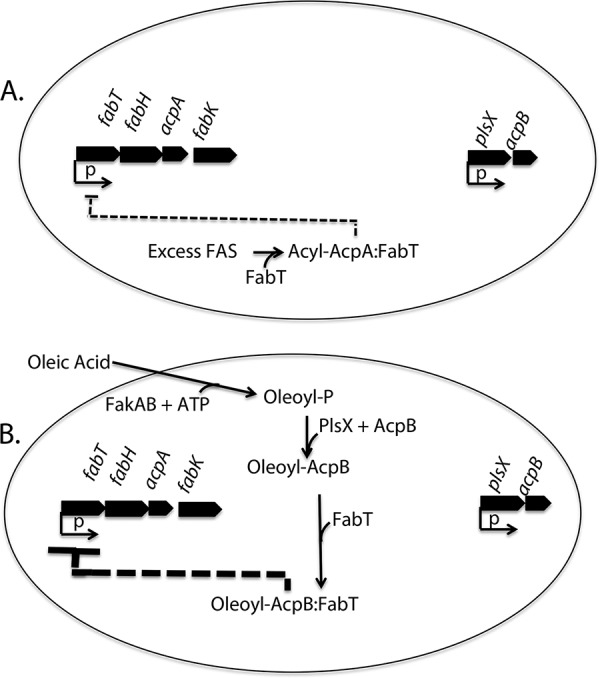
Regulation of fatty acid synthesis by acyl-ACPs. For simplicity, the *fab* and *acc* genes located downstream of *fabK* (see Fig. 1B) and the reactions whereby acyl chains are transferred into phospholipids (Fig. 1B) are not shown. The ovals represent cells grown in the absence (A) or presence (B) of oleate. In the absence of oleate (A), when excess fatty acid synthesis produces more acyl-AcpA than can be consumed in phospholipid synthesis, the excess acyl-AcpA binds FabT and the complex modestly represses transcription of *fabT*, *fabH*, and *acpA* and of the downstream genes. When phospholipid synthesis becomes limited for acyl-AcpA, the acyl-AcpA–FabT complex is disrupted by transfer of the acyl chain into phospholipids, thereby fully restoring *fab* operon transcription. (B) The *acpB* gene continues to be transcribed in the presence of oleate and provides AcpB for conversion to oleoyl-AcpB. The exogenous acid is converted to oleoyl-AcpB via FakAB and PlsX. Oleoyl-AcpB then binds FabT and severely represses *fab* operon transcription. Note that both *fabT* and *acpA* are transcribed under the control of the acyl-AcpA–FabT complex, which, in the absence of AcpB, would result in the futile repression-derepression cycle addressed in the Discussion. Upon exhaustion of the exogenous oleate supply, oleoyl-AcpB would dissociate from FabT and be consumed in phospholipid synthesis, resulting in restoration of fatty acid synthesis.

AcpB and FabT seem almost completely dependent on one another in that essentially the same phenotypes are seen upon deletion of either gene; *de novo* fatty acid synthesis proceeds in the presence of exogenous oleic acid. The most straightforward explanation for these results is that oleoyl-AcpB binds FabT, where it is a more potent regulatory ligand than oleoyl-AcpA (this hypothesis remains to be tested) (Fig. 9). Indeed, without AcpB (and hence without acyl-AcpB), FabT seems unable to repress transcription of the *fab* operon. We found that AcpB is a much better acceptor of acyl chains from acyl-phosphates than is AcpA. Moreover, in the reverse reaction, i.e., transfer of acyl chains from ACP to phosphate, AcpB is much less active than AcpA. These data indicate that the two ACPs interact differently with PlsX and that AcpB seems designed to channel exogenous acyl groups into acyl-AcpB at the expense of acyl-phosphate. The role of channeling may give consistent repression of the *fab* operon during long-term exposure to exogenous fatty acids and thereby avoid a futile cycle resulting from expression of both FabT and AcpA being regulated by FabT and its acyl-ACP ligand.

Consider the case where AcpA would be the sole E. faecalis ACP; oleate addition would shut off synthesis of both AcpA and FabT, and, upon continued growth, the levels of both proteins would decline to the point where repression would be lost. Loss of repression would trigger transcription of the genes of the fatty acid synthesis operon, resulting in restoration of AcpA and FabT levels. This would allow repression to return but at the cost of synthesis of the mRNA and enzymes encoded by the 10 downstream *fab* genes (Fig. 1B) during the intervals when repression was temporarily lifted. This would be wasteful because the fatty acids required for membrane lipid synthesis are provided by the exogenous source and their presence precludes the need for *de novo* fatty acid synthesis. Indeed, the modest deficiency in exogenous fatty acid incorporation of the *ΔacpB* strain may be due to competition for assimilation into phospholipids by acyl-AcpA and acyl-phosphate species synthesized *de novo* during the periods when AcpA and FabT have reached sufficiently low levels to allow a burst of *fab* gene expression. In this scenario, AcpB is not essential because E. faecalis can tolerate the futile cycle without a detectable loss of fitness in the laboratory. However, when AcpB is present (as in wild-type strains), oleoyl-AcpB would bind FabT and stably repress the fatty acid synthesis operon (Fig. 9). Upon exhaustion of the exogenous oleate, the oleyl-AcpB would be consumed in phospholipid synthesis (either directly or indirectly via conversion to oleoyl-phosphate), thereby relieving repression of the fatty acid synthesis operon (Fig. 9). This scenario predicts that a strain lacking PlsX would be defective in repression of fatty acid synthesis because exogenous oleate would not be converted to oleoyl-AcpB, the FabT regulatory ligand. Indeed, a S. pneumoniae *ΔplsX* strain grown with oleate incorporates [^14^C]acetate into phospholipids at about 40% of the level seen in the absence of oleate. This is in marked contrast to the essentially complete inhibition of fatty acid synthesis seen in oleate-grown wild-type strains (23).

An open issue is whether or not AcpB plays a role in lipid synthesis *per se*. The protein carries the needed phosphopantetheine moiety and can be acylated by AasS, an enzyme reported to most active on the ACPs of fatty acid synthesis (24). Moreover, *trans*-2-decenoyl AcpB is a substrate for both E. faecalis enoyl-ACP reductases. However, there are caveats to these observations. AasS is a foreign enzyme, and enoyl-ACP reductases are often active with nonphysiological enoyl-CoA substrates (25). Indeed, enoyl-CoA reduction activity was previously demonstrated for E. faecalis FabI (13). Our inability to delete the *acpA* gene in the presence of oleate supplementation suggests that AcpB cannot efficiently replace AcpA in some step of membrane lipid synthesis. Since fatty acid synthesis is shut down in the presence of oleate, it seems likely that that step(s) would be in incorporation of acyl chains into phospholipids. Incoming oleate would be converted to oleoyl-phosphate by the FakAB kinase, which PlsY would use to acylate *sn*-glycerol-3-phosphate (Fig. 1A) (5, 6). The second acylation, conversion of 1-oleoyl-*sn*-glycerol-3-phosphate to phosphatidic acid by PlsC, requires an acyl-ACP substrate (5, 6), and hence, this acylation may be the step that requires AcpA in E. faecalis, but this requires testing. However, AcpB homologues in other *Lactobacillales* seem likely to function in this acylation reaction. Lactobacillus johnsonii requires fatty acid supplementation for growth (26) and lacks all genes encoding fatty acid synthesis proteins except those encoding AcpS and an AcpB homologue that is 53% identical to E. faecalis AcpB (27). Since the putative *acpB* gene is located immediately downstream of *plsX*, it seems likely that this AcpB homologue functions in 1-oleoyl-*sn*-glycerol-3-phosphate acylation in this bacterium.

Interestingly, *fabT* mutants have been reported to arise during streptococcal infections (28, 29). For example, in a massive genomic sequencing study of 2,954 S. pyogenes strains recovered from infections, fully half of the strains with altered genomes contained mutations in *fabT* (28). Moreover, deletion of *fabT* resulted in a S. pyogenes strain of decreased virulence (28), which raises the issue of whether or not *acpB* mutations might have a similar infection phenotype.

## MATERIALS AND METHODS

### Strains, plasmids, primers, materials, and procedures.

The strains and plasmids used in this study are listed in Table 1. The PCR primers used in this study are presented in Table S1 in the supplemental material. Detailed descriptions of the materials and strain construction and the protein purification and protein modification procedures used in this study are provided in Text S1 in the supplemental material.

**TABLE 1:** Strains and plasmids

Strain or plasmid	Description^*a*^	Reference or source
Strains		
E. coli CY2211	*ΔacpP ΔlacIZYA ΔpanD*::*cat*; pCY765 (*paraBAD*: *acpP*); pCY947 (*placI*^q^ *lacI*:*sfp lacI*^q^)	9
E. coli CY2156	Δ*acpP*::*cat* Δ*lacIZYA*; pCY765 (*paraBAD*: *acpP*); pCY948 (*placI*^q^ *lacI*)	9
E. coli SW158	Φ(*acpP*-Strep tag) (Hyd) (Cm^r^) derivative of MC1061	Laboratory strain
E. coli Rosetta	*ompT hsdS*_B_(r_B_^−^ m_B_^−^) *gal dcm* (DE3) pRARE (Cm^r^)	Novagen
E. coli ZL300	CY2156 carrying pKK233-2	This work
E. coli ZL298	CY2156 carrying pZL298	This work
E. coli ZL299	CY2156 carrying pZL299	This work
E. faecalis FA2-2	Wild type	Laboratory store
E. faecalis ZL317	*acpB* single-crossover strain	This work
E. faecalis ZL318	Δ*acpB*	This work
E. faecalis ZL319	Δ*acpB*	This work
E. faecalis ZL246	Δ*cfa*	This work
E. faecalis ZL115	*fabT* single-crossover strain	This work
E. faecalis ZL116	Δ*fabT*	This work
E. faecalis ZL255	Δ*fabI ΔfabK*	12
E. faecalis ZL279	FA2-2 with *fabT* expression plasmid	This work
E. faecalis ZL303	ZL116 with *fabT* expression plasmid	This work

Plasmids		
pBVGh	Temperature-sensitive β-galactosidase erythromycin‐resistant gene modification vector	33
pKK233-2	*tac* promoter expression vector	Laboratory stock
pBM02	Shuttle vector, E. faecalis expression	34
pZL298	E. faecalis *acpA* expression plasmid derived from pKK233-2	This work
pZL299	E. faecalis *acpB* expression plasmid derived from pKK233-2	This work
pZL311	E. faecalis *acpS* expression vector	This work
pYFJ84	V. harveyi *aasS* expression vector	18
pZL68	E. faecalis *fabI* expression vector	13
pZL72	E. faecalis *fabK* expression vector	13
pZL391	E. faecalis *plsX* expression vector	This work
pZL166	E. faecalis *acpA* expression vector	This work
pZL167	E. faecalis *acpB* expression vector	This work
pTara	Phage T7 polymerase expression	35
pZL276	E. faecalis *acpB* knockout cassette (Cm^r^) in vector pBVGh	This work
pZL114	E. faecalis *fabT* knockout cassette (no resistance marker) in vector pBVGh	This work
pZL234	DNA fragment (400 bp) from E. faecalis *cfa* in pBVGh vector	This work
pZL277	Shuttle plasmid vector with a P32 promoter	This work
pZL278	E. faecalis *fabT* in pZL277	This work

a Cm^r^, chloramphenicol resistance.

10.1128/mBio.00577-19.1 TEXT S1 Detailed description of the materials, detailed strain constructions, and the protein purification and protein modification procedures. Download Text S1, PDF file, 0.1 MB. Copyright © 2019 Zhu et al. 2019 Zhu et al. This content is distributed under the terms of the Creative Commons Attribution 4.0 International license.

10.1128/mBio.00577-19.2 TABLE S1 The PCR primers used in this study. Download Table S1, PDF file, 0.05 MB. Copyright © 2019 Zhu et al. 2019 Zhu et al. This content is distributed under the terms of the Creative Commons Attribution 4.0 International license.

### Gene expression analysis by real-time reverse transcription-quantitative PCR (RT-qPCR).

Total RNA preparations were isolated from the mid-log-phase cells of E. faecalis strains grown in M17 medium by using an RNeasy bacterial RNA isolation kit (Qiagen) as described previously (12). RNA concentrations were determined by using a NanoDrop 2000C spectrophotometer (Thermo Scientific). The cDNA synthesis was performed with an Omniscript reverse transcription (RT) kit (Qiagen). The RT-qPCR assay was conducted using iQ SYBR green Supermix (Bio-Rad) with the 16S RNA gene as an internal control.

### Analysis of phospholipid fatty acids.

The E. faecalis strains were cultured in M17^minus^ medium and labeled with a radioactive acid as follows. For assay of *de novo* synthesis fatty acids, the strains were incubated and grew from an optical density at 600 nm (OD_600_) of 0.05 to an OD_600_ of 0.3 with or without 100 μM oleate. The cultures were then incubated for another 5 h at 37°C in the presence of [1-^14^C]acetate (final concentration of 1 μCi/ml). To assay incorporation of exogenous free fatty acids into phospholipids, the strains were grown from an OD_600_ of 0.1 in the presence of [1-^14^C] oleate (final concentration, 0.1 μCi/ml) with 90 μM nonradioactive oleate for 6 h at 37°C. Cultures were normalized to equal cell concentrations, and the cells were washed thrice with phosphate-buffered saline and then lysed with methanol-chloroform (2:1) solution. The phospholipids were further extracted with chloroform and dried under nitrogen. The fatty acyl groups on phospholipids were then converted to their methyl esters by transesterification with sodium methoxide, extracted into petroleum ether, taken to dryness under nitrogen, resuspended in hexanes, and loaded onto silver nitrate thin-layer chromatography (TLC) plates (Analtech) which were developed in toluene at −20°C (inclusion of silver allows separation of saturated and unsaturated esters). The plates containing the ^14^C-labeled esters were analyzed by phosphorimaging using a GE Typhoon FLA700 scanner and analyzed using the ImageQuant TL program.

For GC-MS analysis, E. faecalis strains were cultured in M17^minus^ medium (with 90 μM oleate) for 6 h at 37°C from an OD_600_ of 0.1. Cultures were standardized, and fatty acid methyl esters were generated as described above and then analyzed by GC-MS using a highly polar chiral CP-Si88 column (Agilent Technologies) as described previously (30). The CP-Si88 column allows baseline separation of the methyl esters of oleic and *cis*-vaccenic acids based on their double-bond positions.

### Quantitation of AcpA and AcpB proteins in E. faecalis.

E. faecalis strains were grown in AC medium with or without 100 μM sodium oleate. The cells were lysed in 25 mM potassium salt-MES (K-morpholineethanesulfonic acid) (pH 6.1) buffer, and the supernatants were loaded onto a 5-ml HiTrap DEAE column. The bound proteins were eluted by the use of 25 mM K-MES (pH 6.1)–2 M KCl solution, heated at 75°C, processed with 50 mM Tris-HCl (pH 10.0)–5 mM dithiothreitol (DTT) buffer for 30 min to deacylate the proteins by cleavage of the thioester bonds, and then dialyzed against a buffer of 50 mM Tris-HCl (pH 8.0) containing 1 mM DTT. The total protein concentrations were adjusted to 40 mg/ml, and 1,600 μg of protein of each extract was transferred into a solution consisting of 0.1 M CoA, 2.5 mM Mg^2+^, 1 mM DTT, and 50 mM Tris-HCl (pH 8.0) at 37°C that contained 5 μM E. faecalis *holo*-ACP synthase (AcpS). After incubation, 1 μM V. harveyi acyl-ACP synthetase (AasS), 2 mM ATP, and 2 μCi/ml [1-^14^C]octanoate were added followed by incubation for 1 h at 37°C. The products were analyzed by the use of 2 M urea–18% PAGE followed by autoradiography.

### Enoyl-ACP reduction by E. faecalis enoyl-ACP reductases.

The synthesized *holo*-AcpA proteins were incubated at 37°C with 1 μM V. harveyi acyl-ACP synthetase (AasS), 2 mM ATP, and 200 μM *trans*-2-decenoic acid to synthesize enoyl-AcpA. The enoyl-AcpA protein was mixed with E. faecalis FabI or FabK at the enzyme-to-substrate ratio of 1:100 in the presence of 2 mM NADH, and the reaction mixture was incubated at 37°C for 3 h. The products were loaded onto 1.5 M urea–18% PAGE conformation-sensitive gels for electrophoresis, and the gels were stained with R-250 Coomassie brilliant blue.

The synthesized *holo*-AcpB protein was incubated at 37°C with 1 μM V. harveyi AasS, 2 mM ATP, and 200 μM *trans*-2-decenoic acid to synthesize *trans*-2-decenoyl-AcpB. The products were recovered by precipitation with 20% trichloroacetic acid (TCA)–0.02% deoxycholate on ice. The precipitants were washed with cold acetone twice and then resuspended in 50 mM Tris-HCl (pH 8.0) buffer. The enoyl-AcpB was mixed with E. faecalis FabI at the enzyme-to-substrate ratio of 1:100 in the presence of 2 mM NADH, and reduction was tracked by measuring the loss of NADH absorption at 340 nm.

### E. faecalis phosphate acyltransferase (PlsX) reactions.

*holo*-ACP (AcpA or AcpB) was incubated at 37°C with 1 μM V. harveyi AasS, 2 mM ATP, and 200 μM long-chain fatty acid (myristic, palmitic, or stearic acids) to synthesize the acyl-ACP species. To measure transfer of the acyl group from ACP to phosphate, the acyl-ACPs were incubated at 37°C with 0.75 mM MgCl_2_, 0.5 mM DTT, 100 mM sodium phosphate, and 0.1 M HEPES (pH 7.0) in the presence of 0.25 μM E. faecalis PlsX. The products were recovered by precipitation on ice with 20% TCA and 0.02% deoxycholate. The precipitants were washed twice with cold acetone, resuspended with 50 mM Tris-HCl (pH 8.0), and loaded onto a 2 M or 2.5 M urea–18% conformationally sensitive gel for electrophoresis. The gels were stained with R-250 Coomassie brilliant blue to detect the proteins.

To measure transfer of acyl groups from acyl-phosphates to *holo*-ACP, incubation was performed at 37°C with 0.05 μM E. faecalis PlsX, 0.75 mM Mg^2+^, 0.5 mM DTT, and myristoyl-phosphate, palmitoyl-phosphate, or stearoyl-phosphate in the presence of 0.1 M HEPES (pH 7.0). The products were recovered and separated by electrophoresis as described above. Long-chain acyl phosphates were synthesized as previously described by Lehninger (31).
