# Supplementary material for: Enterococcus faecalis Encodes an Atypical Auxiliary Acyl Carrier Protein Required for Efficient Regulation of Fatty Acid Synthesis by Exogenous Fatty Acids
Source: mBio. 2019 May 7;10(3):e00577-19. doi: 10.1128/mBio.00577-19 (PMC6509188; doi:10.1128/mBio.00577-19)
Supplement: TEXT S1 [file mBio.00577-19-s0001.pdf]

Supplemental Text

Enterococcus faecalis encodes an atypical auxiliary acyl carrier protein required for efficient regulation of fatty acid synthesis by exogenous fatty acids.

Lei Zhu, Qi Zou, Xinyun Cao, John E. Cronan

Materials

Fatty acids, NADH and antibiotics were purchased from Sigma. Thermo-Fisher provided molecular biology reagents. Qiagen provided DNA purification kits. Sodium [1-^{14}C]acetate (specific activity, 50 mCi/mmol) and [1-^{14}C]oleate (specific activity, 50 mCi/mmol) were purchased from American Radiolabeled Chemicals. Invitrogen provided Ni-agarose columns. GE Healthcare provided the HiTrap Q strong anion-exchange column and Bio-Rad provided the Quick Start Bradford dye reagent. All other reagents were of the highest available quality. Oligonucleotide primers were synthesized by Integrated DNA Technologies and DNA sequencing was done by AGCT.

Construction of *E. faecalis acpB, fabT and cfa* deletion strains

The *E. faecalis ΔacpB* deletion strain was constructed using the protocol described in reference (1) and the PCR primers of Table S1. A cassette for deletion of the gene was constructed by overlap PCR and consisted of chromosomal segments 500 bp upstream and 500 bp downstream of the *acpB* gene coding sequence that bracketed an XbaI site. The chloramphenicol resistance from vector pBM02 obtained by PCR was then inserted into the XbaI site. The assembled construct was then inserted into the PstI and SacI sites of the temperature-sensitive insertion vector pBVGh to give plasmid pZL276 which was transformed into competent cells of *E. faecalis* FA2-2 by electroporation. Transformants were selected on M17 agar medium containing 5 µg/ml of erythromycin at 30°C. One transformant colony was cultured in liquid medium containing erythromycin at 42°C and the cultures were plated on M17 agar medium containing 5

μg/ml erythromycin and 100 μg/ml 5-bromo-4-chloro-3-indolyl-β-D-galactopyranoside (X-Gal) and incubated overnight at 42°C to screen for blue colonies indicating chromosomal integration of the plasmid. One such single crossover strain called ZL317 was cultured in M17 liquid medium containing oleate for 4 h at 30°C and then shifted to 42°C overnight. This step was repeated several times and the final culture was diluted and plated on M17 agar containing X-Gal and oleate and then incubated for 24–48 h at 42°C. Genomic DNA from white colonies, which represent double-crossover events, was extracted and screened by PCR analysis to give the Δ*acpB* strains (named ZL318 and ZL319). Note that although the final strains were resistant to 10 μg/ml chloramphenicol attempts early in the construction to select the single crossover construct by selection for chloramphenicol resistance were unsuccessful perhaps due to phenotypic lag in expression of the cassette.

The *E. faecalis fabT* deletion strain (Δ*fabT*) was constructed similarly to the Δ*acpB* strain except that the deletion cassette did not contain the chloramphenicol resistance cassette. Upstream and downstream 500 bp DNA fragments were assembled by overlap PCR and then inserted into vector pBVGh using NcoI and SacI enzymes site to give pZL144. The Δ*fabT* gene deletion strains without any additional selection markers was constructed and verified as was the Δ*acpB* strains. In this process the single crossover strain was ZL115 and the Δ*fabT* strain was ZL116.

The *fabT* expression plasmid pZL278 was constructed by inserting the *fabT* gene together with a P32 promoter (2) into the shuttle plasmid vector pZL277. Transformation of *E. faecalis* wild type with pZL278 gave strain ZL279 whereas the Δ*fabT* strain gave strain ZL303.

The *E. faecalis cfa* mutation strain was constructed through interruption of the gene by single crossover recombination (3). An internal 409 bp DNA fragment of the *cfa* gene (nucleotides 383 to 791 within the ORF) was inserted into vector pBVGh using PstI and SacI sites to give pZL234. pZL234 was transformed into competent cells of *E. faecalis* FA2-2 and the transformants were selected on GM17 agar medium containing 5 μg/ml of erythromycin at 30°C. One transformant colony was cultured in liquid medium containing erythromycin at 42 °C and the cultures were plated on GM17 agar medium containing 5 μg/ml erythromycin and 100 μg/ml X-Gal and incubated overnight at 42 °C and the Δ*cfa* mutant strain (ZL246 which gives blue colonies on X-Gal plates) were obtained. In this strain, the *cfa* gene ORF is divided to two incomplete segments (ORF nucleotides 1-791 and 383-1167).

β-[^{3}H]Alanine labeling of ACPs in *E. coli*

Strain CY2211 was transformed by the *acpA* or *acpB* gene encoding in pKK233-2 plasmid derivative. Labeling with β-[2,3-3H]alanine in the presence of IPTG in minimal medium was done at 37 °C for 6 h as described previously (4, 5). Cultures were normalized to equal cell concentrations and harvested by centrifugation, resuspended in 100 mM sodium 2-(N-morpholino) ethanesulfonic acid (MES) buffer (pH 6.1) and lysed by sonication. The protein concentrations of the supernatant soluble cell extracts were quantitated using the Bio-Rad protein assay kit. Equal amounts of protein from each sample were analyzed by 20% native polyacrylamide gel (PAGE) followed by radioautography. The gel was soaked in NRAMP100V (Ambion) and dried before analysis.

Expression and Purification of *E. faecalis* AcpA

Plasmid pET28b-*acpA* and the pTara phage T7 polymerase expression plasmid were transformed into *E. coli* strain SW158. The transformants were cultured in LB medium at 37°C with 50 μg/ml kanamycin and 30 μg/ml chloramphenicol to OD_{600} of 0.6 and then induced by 0.2% arabinose and 1 mM IPTG for an additional 4 h. The cells were harvested and lysed in 25 mM K-MES (pH 6.1) buffer. The supernatant was loaded onto the 5 ml Hitrap DEAE column. The bound proteins were eluted with 25 mM K-MES (pH 6.1) containing 2 M KCl and heated at 75°C. The supernatant was then repeatedly loaded onto the Strep-tactin affinity purification column until no *E. coli* Strep-tagged ACP eluted. The eluates were concentrated and then further treated with 85% ammonium sulfate (where ACPs are soluble) and the supernatant was diluted with water and dialyzed against 25 mM K-MES (pH 6.1) buffer and stored at -80°C. The final intact product was analyzed on by liquid chromatography/ electrospray ionization time of flight mass spectroscopy using a Thermo Q Exactive HF-X Hybrid Quadrupole-Orbitrap mass spectrometer.

Expression and Purification of *E. faecalis* AcpB

The pET28b-*acpB* plasmid was transformed into the *E. coli* Rosetta strain. The transformants were cultured in LB medium at 37°C with 50 μg/ml kanamycin and 30 μg/ml chloramphenicol to OD_{600} of 0.6 and then induced with 1 mM IPTG for another 4h. The cells were harvested and

lysed in 25 mM K-MES (pH 6.1) and loaded onto Vivapure D Maxi H DEAE column and the bound proteins were eluted with 25 mM K-MES (pH 6.1) containing 0.25 M KCl. The eluted proteins were dialyzed against 25 mM K-MES (pH 6.1) buffer and stored at -80°C.

Expression and Purification of His_6 tagged proteins

Plasmids derived from pET28b were transformed into the *E. coli* Rosetta strain. The transformants were incubated in LB medium at 37°C with 50 µg/ml kanamycin and 30 µg/ml chloramphenicol to OD of 0.6 and then were induced by 1 mM IPTG for another 4 h incubation. The cells were harvested and lysed in lysis buffer (50 mM sodium phosphate (pH 8.0) 300 mM NaCl, 1 mM DTT and 10 mM imidazole). The supernatant was loaded onto the Ni-NTA column. The column was eluted with a wash buffer (50 mM sodium phosphate (pH 8.0) 300 mM NaCl,1 mM DTT, 40 mM imidazole). and then the tagged proteins were eluted with the same buffer containing 250 mM imidazole. The eluted proteins were dialyzed against 50 mM sodium phosphate (pH 8.0) 300 mM, NaCl, 1 mM DTT, 20% glycerol buffer and stored at -80

Synthesis of *Holo*-AcpA and *Holo*-AcpB

AcpA or AcpB proteins (100 µM) were incubated with 5 µM *E. faecalis holo*-ACP synthase (AcpS), 0.1 M CoA, 2.5 mM $MgCl_2$, 1 mM DTT, 50 mM Tris-HCl (pH 8.0) at 37°C. The products were loaded onto the 0.5 M urea conformationally- sensitive gels for electrophoresis and the gels were stained with R-250 Coomassie Brilliant Blue.

Supplemental Refereences

1. Blancato VS, Magni C. 2010. A chimeric vector for efficient chromosomal modification in *Enterococcus faecalis* and other lactic acid bacteria. Lett Appl Microbiol 50:542-546.

2. Zhu D, Liu F, Xu H, Bai Y, Zhang X, Saris PE, Qiao M. 2015. Isolation of strong constitutive promoters from *Lactococcus lactis* subsp. *lactis* N8. FEMS Microbiol Lett 362: fnv107

3. Bi H, Zhu L, Wang H, Cronan JE. 2014. Inefficient translation renders the *Enterococcus faecalis* FabK enoyl-acyl carrier protein reductase phenotypically cryptic. J Bacteriol 196:170-179.

4. De Lay NR, Cronan JE. 2007. *In vivo* functional analyses of the type II acyl carrier proteins of fatty acid biosynthesis. J Biol Chem 282:20319-20328.

5. Zhu L, Cronan JE. 2015. The conserved modular elements of the acyl carrier proteins of lipid synthesis are only partially interchangeable. J Biol Chem 290:13791-13799.
