# Supplementary material for: Enterococcus faecalis Encodes an Atypical Auxiliary Acyl Carrier Protein Required for Efficient Regulation of Fatty Acid Synthesis by Exogenous Fatty Acids
Source: mBio. 2019 May 7;10(3):e00577-19. doi: 10.1128/mBio.00577-19 (PMC6509188; doi:10.1128/mBio.00577-19)
Supplement: TABLE S1 [file mBio.00577-19-st001.pdf]

Table S1. Oligonucleotide PCR primers

Primers*	Sequence 5’-3’	Utility
efacpA HindIII	actgaagcTTAGTTTGCTTGTTGTTTTTC	*acpA* PCR
efacpA NcoI	ctgaccATGGTATTTGAAAAAATTCAAG	
efacpB NcoI	ctgaccATGGTATTGACTCGTGAAGAAGTACTTC	*acpB* PCR
efacpB HindIII	agctaagcTTATGAATTACTCACAATATAATC	
efacpB up SacI	ATTAAgagctcTAGCTGACGTGGTGGTGACAG	*acpB* L PCR
efacpB up XbaI	ATTATTTTTTAtctagaCAACTGTATTCACCTCCACTG	
efacpB down XbaI	ACAGTTGtctagaTAAAAAATAATAAGACAAAGTCG	*acpB* R PCR
efacpB down PstI	AAAATTctgcagGTAATTTTGGTATGATTGGG	
efAcpS NdeI	ctagcatATGATAAAGGGAATTGGTATTG	*acpS* PCR
efAcpS HindIII	actgaagcTTATTCGCTTTCTAAAACAATTTG	

efplsX for NdeI	ctagcatATGAAAATTGCTGTAGATGC	*plsX* PCR
efplsX Rev Hind III	atgcaagcTTACTCTGCTTTGCCTTCATA	
effabT NdeI	gcatcatATGGTGCACAGAATGGAACC	*fabT* PCR
effabT EcoRI	gatcgaattcTTATTTGTATTCTTGCAAGAAATC	
efCfa inside pstI	ATCGCTGCAGTTAGGCGCTAAATTCCGCTG	409 bp in *cfa*
efCfa inside sacI	ATGCGAGCTCGGCTTGACCAAACAATGACG	
effabT up SacI	TGTATTGTAAGTTTTGATAAATC	*fabT* L arm PCR
effabT up XbaI	CTCACTTTtctagaCCATATACTTTGATTATC	
effabT down XbaI	GTATATGGtctagaAAAGTGAGGATCCCCATGAAG	*fabT* R arm PCR
effabT down PstI	GCCCCATCACCGAAAAGCAC	
Cm For XbaI	gaattccatatgtctagaGTCGACGGCAATAGTTAC	Cm^R PCR
Cm Rev XbaI	aagcttgtcgactctagaCATATGATCCTGGAGCTGTAAT	

*The primer sequences were based on the *E. faecalis* V583 genome. Lower case type denotes restriction sites.
